# Collagen proteins are found also within the neural parenchyma in the healthy CNS

**DOI:** 10.3934/Neuroscience.2021019

**Published:** 2021-03-16

**Authors:** Arosh S Perera Molligoda Arachchige

**Affiliations:** Department of Biomedical Sciences, Humanitas University, Milan, Italy

To the Editor:

In a recent News & Views article published in *Nature Neuroscience* Michael V. Sofroniew [1] claims that in healthy CNS, collagen-producing cells and collagen protein are present only along blood vessels and meninges, and they are not found within the neural parenchyma. However, we do not agree with this statement. Whilst, it is true that collagen-producing cells and collagen protein are found in blood vessels and meninges within the healthy CNS, we found evidence in the broader literature to show that collagen proteins are present also within the neural parenchyma. For example, Gregorio et al. [2] in their review have described the presence of collagen VI protein in the neural parenchyma in the healthy CNS. Furthermore, a study by Zech et al. [3] revealed the presence of Col6a3 mRNA in neurons throughout the adult mouse brain, even in the absence of injury. Moreover, Roggendorf et al. [4] in one of their studies using monospecific affinity-purified polyclonal antibodies against collagen VI by applying peroxidase-anti peroxidase- and alkaline phosphatase-anti alkaline phosphatase techniques, have noted intensive staining in the superficial glia, choroid plexus, and sheath of cranial nerves.

Apart from this, the ECM also exists in the nervous tissue itself, in the form of perineuronal nets and the neural interstitial matrix dispersed in the parenchyma [5] , which the author has not discussed. This is supported by evidence from a study conducted by Su et al. [6] where the loss of interneuron-derived collagen XIX led to a reduction in perineuronal nets in the mammalian telencephalon which may contribute to complex brain disorders, including schizophrenia. The author seems to have missed recent studies indicating the role of collagens in the developing CNS parenchyma such as guiding synaptogenesis, axonal guidance, and Schwann cell differentiation [7]. Indeed, also Dziadek et al. [8] have observed Collagen VI in the mesenchyme of the developing murine choroid plexus at E14 (embryonic day 14). Based on the facts addressed above, we conclude that the author could have made that statement if it had been made clear that there exist exceptions to that affirmation, which in this case could have been supported by the observation of both collagen VI and XIX in healthy neural parenchyma.
